# Differential expression of long-term depression, and synaptic tagging and capture in mouse hippocampal area CA2 synapses

**DOI:** 10.1093/pnasnexus/pgaf241

**Published:** 2025-07-29

**Authors:** Zijun Wang, Lik-Wei Wong, Sreedharan Sajikumar

**Affiliations:** Department of Physiology, Yong Loo Lin School of Medicine, National University of Singapore, Singapore 117597, Singapore; Life Science Institute Neurobiology Programme, National University of Singapore, Singapore 117456, Singapore; Department of Physiology, Yong Loo Lin School of Medicine, National University of Singapore, Singapore 117597, Singapore; Life Science Institute Neurobiology Programme, National University of Singapore, Singapore 117456, Singapore; Department of Physiology, Yong Loo Lin School of Medicine, National University of Singapore, Singapore 117597, Singapore; Life Science Institute Neurobiology Programme, National University of Singapore, Singapore 117456, Singapore; Healthy Longevity Translational Research Programme, Yong Loo Lin School of Medicine, National University of Singapore, Singapore 117456, Singapore

**Keywords:** CA2 region, long-term depression, synaptic tagging and capture, CPLX2, p75^NTR^

## Abstract

CA2 hippocampal neurons have received renewed interest due to their unique functions and plasticity properties that differ between synapses within the same neuronal population. However, detailed studies on long-term depression (LTD) in CA2 pyramidal neurons are lacking. In this study, LTD was induced and characterized at both Schaffer collateral-CA2 (SC-CA2) and entorhinal cortex-CA2 (EC-CA2) synapses in young, male mice. This LTD was found to be dependent on N-methyl-D-aspartate receptors, protein synthesis, and p75 neurotrophin receptors. However, weaker stimulations could only induce early LTD in EC-CA2 but not SC-CA2 synapses, consistent with its “plasticity-resistant” nature. CA2 LTD is capable of undergoing heterosynaptic synaptic tagging and capture (STC), although the machinery involved differs between SC-CA2 and EC-CA2 synapses. SC-CA2, but not EC-CA2, requires precursor brain-derived neurotrophic factor activity to maintain LTD. Subsequently, quantitative shotgun proteomics analysis yields complexin-2 as a strong candidate plasticity-related product involved in LTD in the CA2. These results reveal interesting differences in STC machinery between synaptic populations of a common set of neurons, enhancing our understanding of hippocampal circuitry involving the CA2. Interesting implications regarding the heterogeneous biochemical makeup of CA2 pyramidal neurons and fundamental STC theory that arise as a consequent of our results are also discussed further.

Significance Statement The hippocampal CA2 area regulates hippocampal activity, an essential correlate for many memory-related cognitive functions, by integrating different information streams. In this study, we characterize the expression and properties of long-term depression within CA2 pyramidal neurons. We find that these neurons have different plasticity and associativity properties between entorhinal cortex and CA3 inputs that represent different information streams. Biochemical analyses suggest that proBDNF and complexin-2 are two candidate proteins correlated with these electrophysiological differences. This study develops our understanding of a key plasticity process essential for flexibility and adaptation, crucial in a modulatory region. It also builds upon the ever complex story of the unique plasticity features of CA2 neurons, with postsynaptic complexin-2 being a previously overlooked biochemical target suitable for further exploration.

## Introduction

The CA2 subfield of the hippocampus is a small region between the CA1 and CA3 that is of research interest due to its recently discovered unique functions and characteristics (1–3). CA2 pyramidal neurons are very well connected to other regions throughout the hippocampus. It receives excitatory signals from the CA3, dentate gyrus, and entorhinal cortex layer II, while projecting to the CA1, CA3, entorhinal cortex, and recurrently back to the CA2 (1, 3, 4). For such a small region to be so well connected suggests that the CA2 likely has distinct and important functions influencing the rest of the hippocampus circuitry (3). Indeed, the CA2 has been found to be necessary for social memory and regulates signaling in other regions within the hippocampus (5, 6).

Synaptic plasticity refers to the ability of synapses to change their connective efficacy in response to activity occurring specifically at the synapse (7). Long-term potentiation (LTP) and long-term depression (LTD) are two well-established forms of synaptic plasticity that are regarded as cellular correlates of memory. LTP refers to an input-specific increase in synaptic efficacy in response to neuronal activity at the synapse, while LTD is similar except that it refers to a decrease instead. LTP and LTD occur in two main phases. The early phase (early LTP/LTD) is protein synthesis independent and only lasts for around 2 h. Should the plasticity-inducing event be sufficiently strong, the late phase occurs (late LTP/LTD), which is typically protein synthesis dependent and can last for more than 24 h in vivo. LTP and LTD show cellular association, and such an association is explained by synaptic tagging and capture (STC) hypothesis (8, 9). Under STC hypothesis, induction of early LTP causes the temporary setting of a synaptic tag within the affected synapse. These synapses can then undergo late LTP due to the synthesis of plasticity-related products (PRPs), an effect of suprathreshold stimulation, and the subsequent capture of these PRPs by the synaptic tag (9). LTP and LTD are capable of heterosynaptic associativity via the STC process, during which PRPs synthesized due to strong stimulation at one set of synaptic inputs can be captured by synaptic tags set at another distinct set of synapses, even if these synapses have only experienced subthreshold activity. This interaction results in the expression of late LTP/LTD at the latter set of synapses (9).

Synaptic plasticity properties of CA2 synapses are highly heterogeneous. It has been shown that while synaptic connections from the entorhinal cortex (EC-CA2) readily undergo LTP, those from the CA3 via the Schaffer collaterals (SC-CA2) do not express LTP as a result of high frequency stimulation (10). This is due to the unique molecular environment of SC-CA2 synapses, which resist plasticity (11). These mechanisms include enhanced calcium dampening, such as via RGS14, and ERK/MAPK signaling cascade inhibition via STEP (12–17). Extracellularly, the presence of perineuronal nets and feedforward inhibition of excitatory signals from the CA3 via parvalbumin-positive interneurons additionally dampens plasticity (18–20). Although numerous mechanisms confer resistance to plasticity, it is not overly difficult to experimentally enable plasticity in SC-CA2 synapses. For instance, SC-CA2 synapses express strong tetanization-induced LTP when RGS14 is knocked out, or when group III metabotropic glutamate receptors are inhibited (14, 21). Plasticity can also be unlocked via priming by neuromodulators such as through cholinergic or serotonergic pathways (22, 23). Considering that these methods only target at most one (or none in the case of neuromodulatory priming) out of the many plasticity resistance mechanisms within the CA2, it is intriguing that the synapses can achieve LTP, while most resistance mechanisms are still active (24).

The expression of associativity via STC is also heterogeneous within the CA2. It is important to note that STC can only occur if the PRPs available at a synapse are compatible for capture by the synaptic tags set at said synapse. Substance P is known to cause activity-dependent slow-onset potentiation (SOP) in both EC-CA2 and SC-CA2 synapses, and this activity dependency can be taken advantage of experimentally to only induce SOP in one set of synapses (25). Interestingly, SOP in SC-CA2 followed by weak tetanization in EC-CA2 results in expression of late LTP in EC-CA2. However, SOP in EC-CA2 does not allow for subsequent expression of LTP in SC-CA2 even with strong tetanus (25). These experiments hint towards the possibility that PRPs produced by SOP via substance P via EC-CA2 may differ from those via SC-CA2, or conversely, that the synaptic tags have differing molecular targets between SC-CA2 and EC-CA2 synapses. It is hence rather likely that plasticity processes in SC-CA2 and EC-CA2 synapses involve different mechanisms or molecular players even beyond the presence of plasticity suppressing pathways.

While significant progress has been made in understanding LTP within CA2 neurons, much less is known about their ability to express LTD. Early reports have suggested that LTD does occur within the CA2, although its expression is inconsistent (10). More recently, Dudek group showed that the CA2 is capable of adenosine receptor-mediated chemical LTD and metabotropic glutamate receptor-dependent (mGluR) LTD (26, 27). However, we could not find any works within the literature dissecting the expression and mechanisms of activity-induced LTD in the CA2. In this study, we aim to explore and characterize the expression of LTD within CA2 pyramidal neurons, comparing between SC-CA2 and EC-CA2 synapses. We then seek to investigate LTD-induced associativity via STC between these two synapses, in particular attempting to define the properties and molecular players involved in such associations. We find that while N-methyl-D-aspartate (NMDA) receptor-dependent LTD is STC compatible and can occur at both SC-CA2 and EC-CA2 synapses, the ease of induction differs. Further in-depth investigation shows that PRPs synthesized and captured as a result of LTD-inducing stimulus at these two synapses differ, with precursor brain-derived neurotrophic factor (proBDNF) being involved with SC-CA2, while complexin-2 (CPLX2) appears to be a candidate with strong potential at EC-CA2 synapses. The findings further support the possibility of heterogeneous biochemical synaptic properties among CA2 pyramidal neurons.

## Results

### Basic properties of LTD in the CA2

**Fig. 1.**
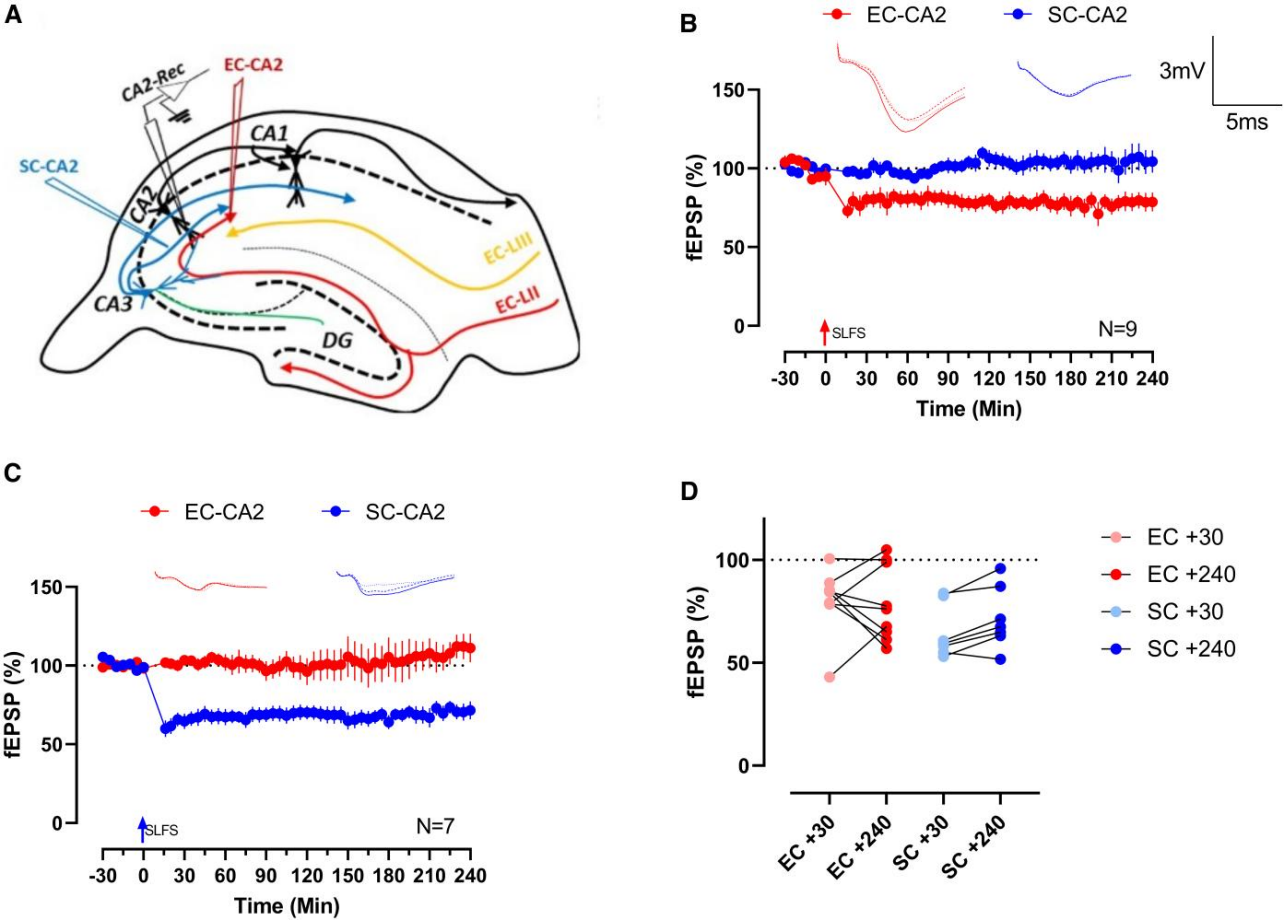
CA2 synapses express LTD. A) Location of electrodes on transverse hippocampal slices. The black recording electrode is placed within the distal dendritic region of the CA2 neurons, flanked by two stimulating electrodes (blue, SC-CA2 stimulating; red, EC-CA2 stimulating). SLFS applied to B) EC-CA2 (red dots; *n* = 9) or C) SC-CA2 (blue dots; *n* = 7) synapses of WT animals leads to long-lasting depression of synaptic strength. Insets depict representative traces at −15 min (solid line), +30 min (dashed line), and +240 min (dotted line). Scale bars for traces represent 3 mV (vertical) and 5 ms (horizontal), respectively, and are common between subfigures. Error bars indicate ± SEM. D) Scatter plot of experiments in B) and C) at two distinct time points resulting in four groups of data: 30 min (pale red) or 240 min (red) after SLFS application to EC-CA2, and 30 min (pale blue) or 240 min (blue) after SLFS application to SC-CA2. Lines join data points measured from the same experiment. Dashed line indicates average baseline values (100% of baseline fEPSP). Experiments where synaptic efficacy reaches or approaches baseline are considered to have failed to establish late LTD. *n* refers to slice replicates. Figure 1A reused from (25).

We first sought to investigate the possibility for LTD expression in the CA2 hippocampal subfield using field electrophysiology. In these experiments, stimulating electrodes are placed at the SC-CA2 and EC-CA2 pathway, respectively, towards a common population of CA2 neurons in which a recording electrode is placed (Fig. 1A). Verification and characterization of the two pathways based on this experimental setup can be found in our previous papers (25, 28). Experimental results are typically compared, when possible, both against baseline recordings (Wilcox test) and the control pathway in a time-matched fashion (*U* test). Application of strong low-frequency stimulation (SLFS, see Materials and methods) typically results in late LTD in SC-CA1 synapses. Applying SLFS via the EC-CA2 pathway results in significant depression (Wilcox test *P* = 0.0039; *U* test *P* = 0.0019; +30 min, Fig. 1B) that lasts for up to 4 h (Wilcox test *P* = 0.0117; *U* test *P* = 0.0315; +240 min, Fig. 1B). Surprisingly, SLFS application to SC-CA2 synapses similarly caused strong depression of synaptic efficacy (Wilcox test *P* = 0.0156; *U* test *P* = 0.0006; +30 min, Fig. 1C) that maintained throughout the experiment (Wilcox test *P* = 0.0156; *U* test *P* = 0.0070; +240 min, Fig. 1C). As there are previous reports that LTD in CA2 neurons is inconsistently induced, we provide a scatter plot of these LTD induction experiments (Fig. 1D), which show that there is a small proportion of experiments on the EC-CA2 in which SLFS application failed to induce LTD. These results are intriguing when considering that SC-CA2 synapses typically are considered plasticity resistant and do not undergo LTP. Note that later experiments focusing solely on the late phase of LTD (i.e. STC and PRP-related experiments) are presented after filtering out samples which failed to induce early LTD.

**Fig. 2.**
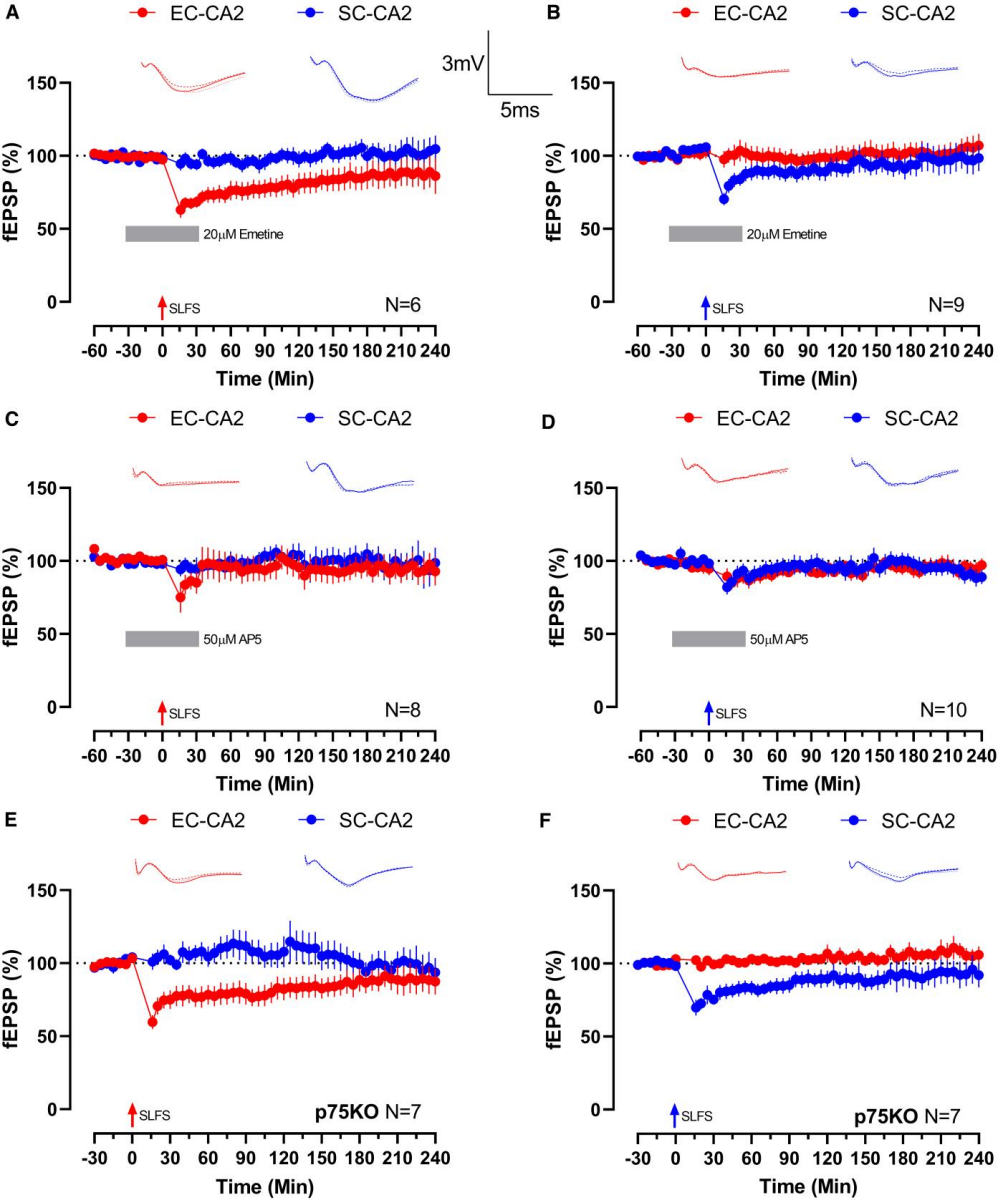
Basic characterization of SLFS-induced LTD in CA2. Application of SLFS to A) EC-CA2 (red dots; *n* = 6) or B) SC-CA2 (blue dots; *n* = 9) in WT animals during acute bath application of protein synthesis inhibitor 20 μM emetine (−30 to +30 min) resulted in a reduction of synaptic efficacy that returned to baseline before the end of the experiment. SLFS delivered to C) EC-CA2 (red dots; *n* = 8) or D) SC-CA2 (blue dots; *n* = 10) of WT animals did not cause substantial changes to fEPSP values throughout the experiment. SLFS application to E) EC-CA2 (red dots; *n* = 7) or F) SC-CA2 (blue dots; *n* = 7) in p75KO animals caused transient depression that did not last for 4 h. Insets depict representative traces at −15 min (solid line), +30 min (dashed line), and +240 min (dotted line). Scale bars for traces represent 3 mV (vertical) and 5 ms (horizontal), respectively, and are common between subfigures. Error bars indicate ± SEM. *n* refers to slice replicates.

We then sought to characterize the basic molecular properties of SLFS-induced LTD in the CA2, in comparison with the typical characteristics of LTD (29). To determine whether this LTD is protein synthesis dependent, SLFS application was performed under the acute influence of protein synthesis inhibitor 20 μM emetine, which was bath applied prior and subsequent to SLFS for a total duration of 1 h (from −30 to +30 min). In these experiments, SLFS applied to EC-CA2 pathway resulted in strong reduction in synaptic strength (Wilcox test *P* = 0.0313; *U* test *P* = 0.0043; +30 min, Fig. 2A) that did not last to the end of the experiment (both tests *P* > 0.05; +240 min, Fig. 2A). Similarly, SLFS applied to SC-CA2 pathway resulted in an immediate depression (Wilcox test *P* = 0.0195; *U* test *P* = 0.0400; +30 min, Fig. 2B) that swiftly returned to baseline (both tests *P* > 0.05; +240 min, Fig. 2A). To distinguish whether SLFS-induced LTD utilized NMDA receptor-dependent or mGluR-dependent mechanisms, we attempted to induce LTD under the acute influence of NMDA receptor antagonist 50 μM AP5 (from −30 to +30 min). SLFS delivered to EC-CA2 (both tests *P* > 0.05; Fig. 2C) and SC-CA2 (both tests *P* > 0.05; Fig. 2D) did not cause substantial change to synaptic efficacy. Lastly, late phase of NMDA receptor-dependent LTD in SC-CA1 synapses critically requires proBDNF/p75^NTR^ interaction (30, 31). To test whether CA2 LTD requires such interaction, we attempted to induce LTD in p75^NTR^ knockout (p75KO) mice. In these experiments, SLFS applied to EC-CA2 synapses resulted in depression (Wilcox test *P* = 0.0156; *U* test *P* = 0.0175; +30 min, Fig. 2E) that did not sustain until the end of the experiment (both tests *P* > 0.05; +240 min, Fig. 2E). SLFS delivered to SC-CA2 synapses similarly caused a reduction in synaptic efficacy (Wilcox test *P* = 0.0156; *U* test *P* = 0.0006; +30 min, Fig. 2F) that was returned to baseline by the end of the experiment (both tests *P* > 0.05; +240 min, Fig. 2F). We also successfully verified that this LTD in p75KO mice has similar properties as LTD in WT mice in terms of protein synthesis and NMDA receptor dependency (Fig. S1). These experiments indicate that SLFS-induced LTD in CA2 pyramidal neurons is dependent on protein synthesis, NMDA receptors, and p75^NTR^, which suggests molecular mechanistic overlap with SLFS-induced LTD at SC-CA1 synapses.

### Associativity of LTD in the CA2

**Fig. 3.**
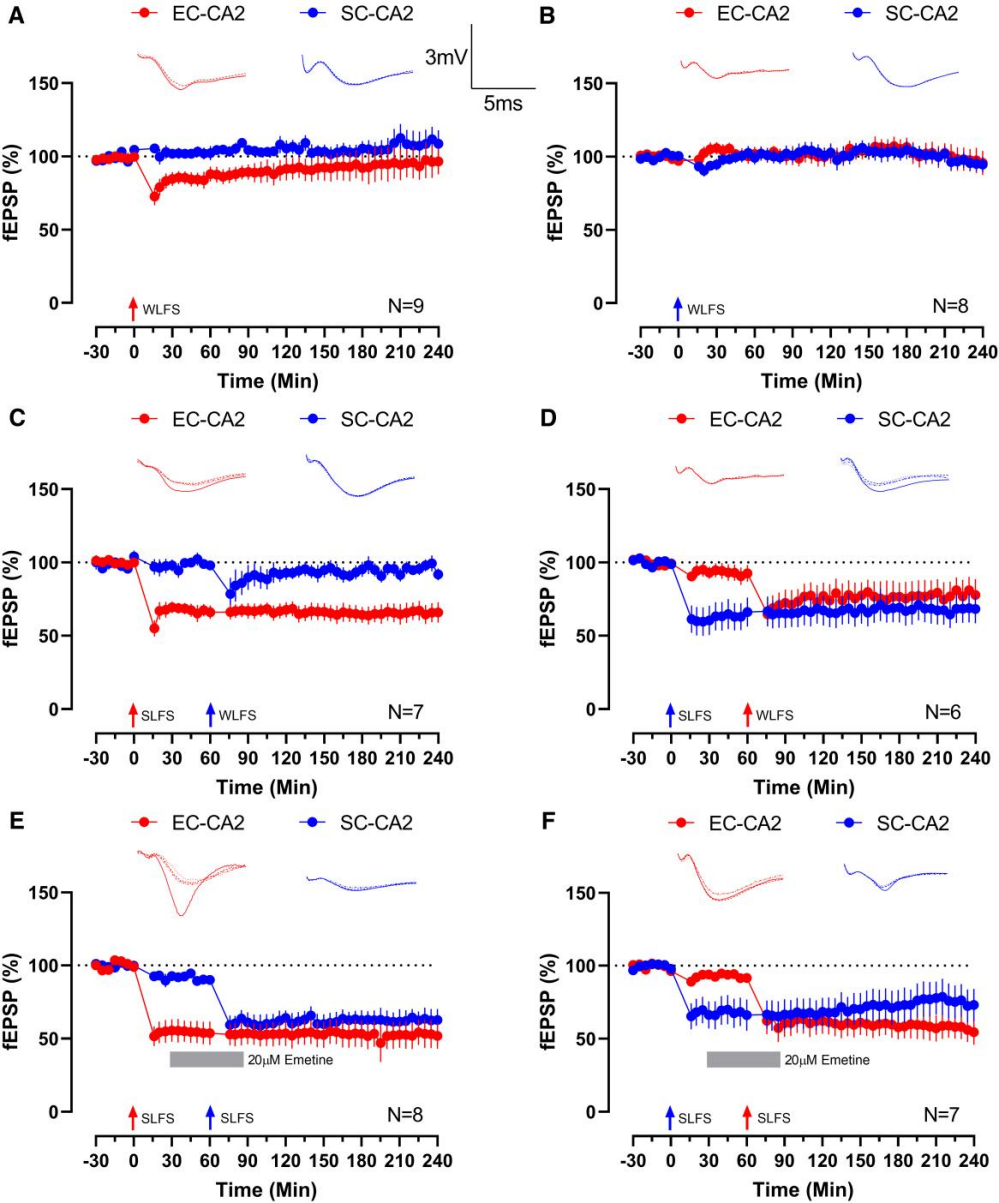
CA2 synapses express different associative properties. A) Application of WLFS to EC-CA2 synapses in WT animals results in a temporary reduction in synaptic strength that swiftly returns to baseline (red dots, *n* = 9). B) WLFS delivered to SC-CA2 synapses in WT animals did not cause significant changes in synaptic efficacy (blue dots, *n* = 8). C) Strong-before-weak experiment in the EC-CA2 to SC-CA2 direction. Application of SLFS to EC-CA2 synapses caused sustained depression as expected (red dots, *n* = 7). Subsequent application of WLFS to SC-CA2 synapses in the same hippocampal slice resulted in no substantial changes to synaptic strength (blue dots, WLFS at +60 min). D) Strong-before-weak experiment in the SC-CA2 to EC-CA2 direction. SLFS application to SC-CA2 synapses caused long-lasting depression consistent with earlier experiments (blue dots, *n* = 6). Subsequent WLFS delivery to EC-CA2 synapses in the same slice results in strong depression that lasted until the end of the experiment (red dots, WLFS at +60 min). E) STC experiments using SLFS under protein synthesis inhibition as a tag-setting only synaptic stimulation. SLFS application to EC-CA2 synapses results in long-lasting reduction of synaptic efficacy as shown in earlier experiments (red dots, *n* = 8). Subsequent SLFS application to SC-CA2 synapses still resulted in sustained depression despite the influence of 20 μM emetine (blue dots; emetine applied from +30 to +90 min, SLFS at +60 min). F) Similar experiment to E) but in the opposite direction. Application of SLFS to SC-CA2 synapses results in diminished synaptic strength that is long-lasting (blue dots, *n* = 7) as expected. Subsequent application of SLFS to EC-CA2 caused expression of sustained depression even in the presence of 20 μM emetine (red dots, emetine applied from +30 to +90 min, SLFS at +60 min). Insets depict representative traces at −15 min (solid line), +30 min (dashed line), and +240 min (dotted line). For STC experiments (C–F), a fourth trace at +90 min (dotted and dashed lines) is provided. Scale bars for traces represent 3 mV (vertical) and 5 ms (horizontal), respectively, and are common between subfigures. Error bars indicate ± SEM. *n* refers to slice replicates.

Under LTP-inducing protocols, CA2 neurons do not display associativity via STC between EC-CA2 and SC-CA2 synapses, in part due to the LTP-resistant nature of SC-CA2 synapses (10, 11). However, it has been difficult to distinguish whether other causes preventing STC are present as interventions that promote SC-CA2 LTP also promote STC (21, 22, 25, 32). Since LTD is possible in both pathways, we can now investigate whether STC is possible independent of the feasibility for homosynaptic plasticity. Experimentally, STC can be observed via the strong-before-weak protocol, where a strong stimulation capable of both tag setting and PRP synthesis is first delivered to one synaptic population, followed by a weak stimulation only capable of tag setting to another pathway (8). Weak low-frequency stimulation (WLFS, see Materials and methods) has been proven to promote only early LTD, and its capacity to do so in CA2 must first be tested before further experiments (29). Application of WLFS to EC-CA2 synapses resulted in depression (Wilcox test *P* = 0.0039; *U* test *P* = 0.0012; +30 min, Fig. 3A) that swiftly returned to baseline (Wilcox test *P* > 0.05; +240 min, Fig. 3A). On the contrary, WLFS application in SC-CA2 synapses did not cause any significant changes in synaptic efficacy (both tests *P* > 0.05; +30 and +240 min, Fig. 3B). A comparison between the two experiments is shown in Fig. S2.

We then investigated whether associativity can occur using the strong-before-weak protocol. SLFS applied to EC-CA2 synapses resulted in long-lasting depression consistent with earlier experiments (Wilcox test *P* = 0.0156; +240 min, red circles, Fig. 3C). Interestingly, subsequent WLFS applied to SC-CA2 synapses did not cause substantial shifts in synaptic strength (Wilcox tests *P* > 0.05; +90 and +240 min, blue circles, Fig. 3C). Interestingly, outcomes differ when attempting association in the reverse direction. SLFS applied to SC-CA2 synapses causes significant reduction in synaptic efficacy consistent with previous experiments (Wilcox test *P* = 0.0313; +240 min, blue circles, Fig. 3D). WLFS subsequently delivered to EC-CA2 synapses also resulted in strong depression (Wilcox test *P* = 0.0313; +90 min, red circles, Fig. 3D) that last for 4 h (Wilcox test *P* = 0.0625; +240 min, red circles, Fig. 3D). These experiments were also repeated in p75KO mice, which expectedly did not express associativity via STC in both directions due to its incapability for late LTD (Fig. S3). Put together, these results indicate that associativity via STC only occurs in the SC-CA2 to EC-CA2 direction, but not the EC-CA2 to SC-CA2 direction.

However, considering that WLFS failed to induce early LTD in SC-CA2, the lack of associativity from EC-CA2 to SC-CA2 could simply be due to the lack of synaptic tags at SC-CA2 after WLFS. This possibility must be eliminated to confirm these observations. Hence, we repeated strong-before-weak experiments, replacing WLFS with SLFS under the acute influence of emetine (+30 to +90 min). Since emetine blocks protein synthesis, LTD induction using SLFS would result only in the production of functional synaptic tags without PRPs. Hence, the cause for STC deficiency, should it still be the case, would likely lie in incompatibilities between PRPs, respectively, produced by SLFS in the two synaptic pathways. In these experiments, initial SLFS (without emetine) in EC-CA2 synapses caused lasting depression similar to earlier experiments (Wilcox test *P* = 0.0078; +240 min, red circles, Fig. 3E). Subsequent SLFS in SC-CA2 synapses under the influence of 20 μM emetine also caused significant reduction of synaptic efficacy (Wilcox test *P* = 0.0078; +90 min, blue circles, Fig. 3E) that maintained throughout the experiment (Wilcox test *P* = 0.0156; +240 min, blue circles, Fig. 3E). In the other direction, SLFS application in SC-CA2 synapses resulted in sustained reduction of synaptic strength (Wilcox test *P* = 0.0313; +240 min, blue circles, Fig. 3F). Subsequent SLFS application in EC-CA2 synapses under the influence of emetine also resulted in long-lasting depression (Wilcox test *P* = 0.0156; +240 min, red circles, Fig. 3F) consistent with the SLFS-before-WLFS experiments (Fig. 3D). The STC process successfully occurred in both directions, which suggests that the previous inability for association between SLFS-induced LTD at EC-CA2 synapses and WLFS-affected SC-CA2 synapses is due to differences in the synaptic tagging process associated with WLFS at SC-CA2 synapses. These differences could have resulted in tags which are incompatible with SLFS-produced PRPs or that the tagging process is deficient. These results also show that CA2 pyramidal neurons are capable of heterosynaptic STC.

### Identification of PRPs involved in LTD in the CA2

**Fig. 4.**
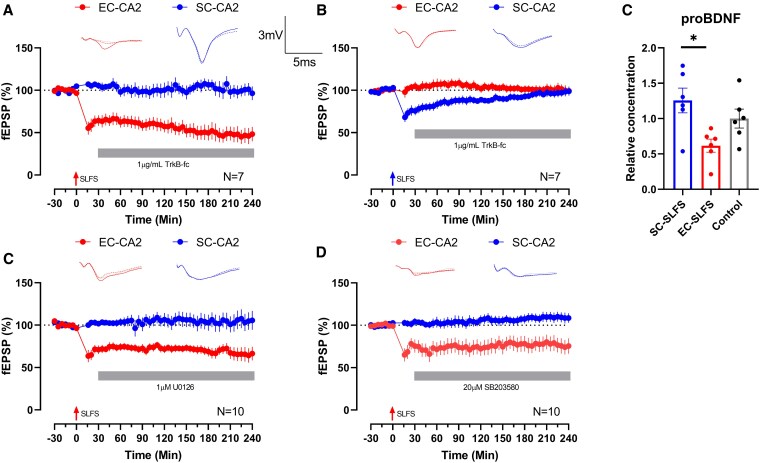
PRPs involved in LTD differ between SC-CA2 and EC-CA2 synapses. These experiments investigate potential PRPs involved in CA2 LTD by testing their necessity for LTD maintenance. A) SLFS applied to EC-CA2 synapses results in long-lasting depression that was not affected by continuous bath application of proBDNF chelator 1 μg/mL TrkB-fc throughout the maintenance phase (red circles, *n* = 7, TrkB-fc from +30 to +240 min). B) Application of SLFS to SC-CA2 synapses instead results in a reduction in synaptic efficacy that could not be sustained under the influence of 1 μg/mL TrkB-fc (blue circles, *n* = 7; TrkB-fc from +30 to +240 min). C) Results from ELISA comparing proBDNF expression between slices collected 60 min after SLFS delivery to SC-CA2 synapses (SC-SLFS, blue points), 60 min after SLFS delivery to EC-CA2 synapses (EC-SLFS, red points), and unstimulated slices (control, black points; all groups *n* = 6). *Asterisks indicate differences of statistical significance (*P* < 0.05) SLFS application to EC-CA2 synapses (red circles) caused long-lasting reduction in synaptic strength that was unaffected by D) ERK1/2 inhibition via 1 μM U0126 (*n* = 10) or E) p38 MAPK inhibition via 20 μM SB203580 (*n* = 10). Both drugs were applied continuously in a similar fashion to TrkB-fc experiments (drugs bath applied from +30 to +240 min). Insets depict representative traces at −15 min (solid line), +30 min (dashed line), and +240 min (dotted line). Scale bars for traces represent 3 mV (vertical) and 5 ms (horizontal), respectively, and are common between subfigures. Error bars indicate ± SEM. *n* refers to slice replicates.

Considering the numerous differences in plasticity properties between EC-CA2 and SC-CA2 synapses, shown so far in this study or in other papers, there are likely interesting differences in molecular mechanisms governing plasticity processes between these two synaptic populations. Hence, we sought to identify whether the PRPs synthesized as a result of SLFS at EC-CA2 or SC-CA2 synapses differ between each other or with other synaptic populations within the hippocampus. Since PRPs are necessary for the maintenance of LTD as it transitions from early LTD to late LTD, pharmacological inhibition of PRPs during the maintenance phase of LTD should prevent the expression of late LTD. proBDNF is known for its importance for LTD via its interaction with p75^NTR^ and could play similar roles as the LTD counterpart for brain-derived neurotrophic factor (BDNF), which is a PRP involved in LTP (30, 33, 34). Application of SLFS to EC-CA2 synapses resulted in sustained strong depression (Wilcox test *P* = 0.0156; *U* test *P* = 0.0041; +240 min, Fig. 4A) even under continuous bath application (+30 to +240 min) of proBDNF chelator 1 μg/mL TrkB-fc chimeric protein (TrkB-fc) throughout the maintenance phase of LTD. Interestingly, SLFS delivered to SC-CA2 synapses resulted in only a temporary reduction in synaptic strength that swiftly returned to baseline (both tests, *P* > 0.05; +240 min, Fig. 4B) under continuous application of TrkB-fc. These data suggest that only maintenance of LTD in SC-CA2 synapses is proBDNF dependent, which suggests that it may be a key PRP involved in LTD at SC-CA2 synapses. To verify this possibility, ELISA was performed using excised CA2 samples collected without stimulation (control), 60 min after SLFS to EC-CA2 synapses (EC-SLFS) or 60 min after SLFS to SC-CA2 synapses (SC-SLFS). proBDNF expression in SC-SLFS tissues is significantly higher than that in EC-SLFS tissues, which suggests that proBDNF is indeed a PRP that contributes uniquely to SC-CA2 LTD but not EC-CA2 LTD (Welch's ANOVA W = 6.004, *P* = 0.0210; Games–Howell's post hoc test *P* = 0.0310; Fig. 4C, *n* = 6 per group). While mature BDNF is also chelated by TrkB-fc, studies have shown greater relative importance of proBDNF over mature BDNF in LTD (30, 35, 36); hence, we focused our efforts on proBDNF only in this study.

Since EC-CA2 and SC-CA2 synapses are capable of association via heterosynaptic STC, there are likely some common PRPs synthesized as a result of SLFS at both synapses for such associativity to be possible. There must also be some proteins involved as PRPs in EC-CA2 synapses for LTD to be possible in the first place. We tested ERK1/2 as it is known for its necessity for late LTD and has shown sustained activity during the maintenance of dopamine-dependent forms of synaptic plasticity (37, 38). SLFS at EC-CA2 synapses caused sustained depression of synaptic efficacy (Wilcox test *P* = 0.0020; *U* test *P* = 0.0279; +240 min, Fig. 4D) even with continuous infusion of ERK1/2 inhibitor U0126 (+30 to +240 min). p38 MAPK (MAPK14) was another candidate PRP chosen for its functional similarity with ERK1/2 and for its involvement in LTD (39–41). Application of SLFS to EC-CA2 synapses resulted in depression throughout the experiment even under continuous influence (+30 to +240 min) of p38 MAPK inhibitor 20 μM SB203580 (Wilcox test *P* = 0.0273; *U* test *P* = 0.0115; +240 min, Fig. 4E). As the activity of ERK1/2 and p38 MAPK are not necessary for the maintenance of LTD at EC-CA2 synapses, they are unlikely to be the PRPs involved in this process.

**Fig. 5.**
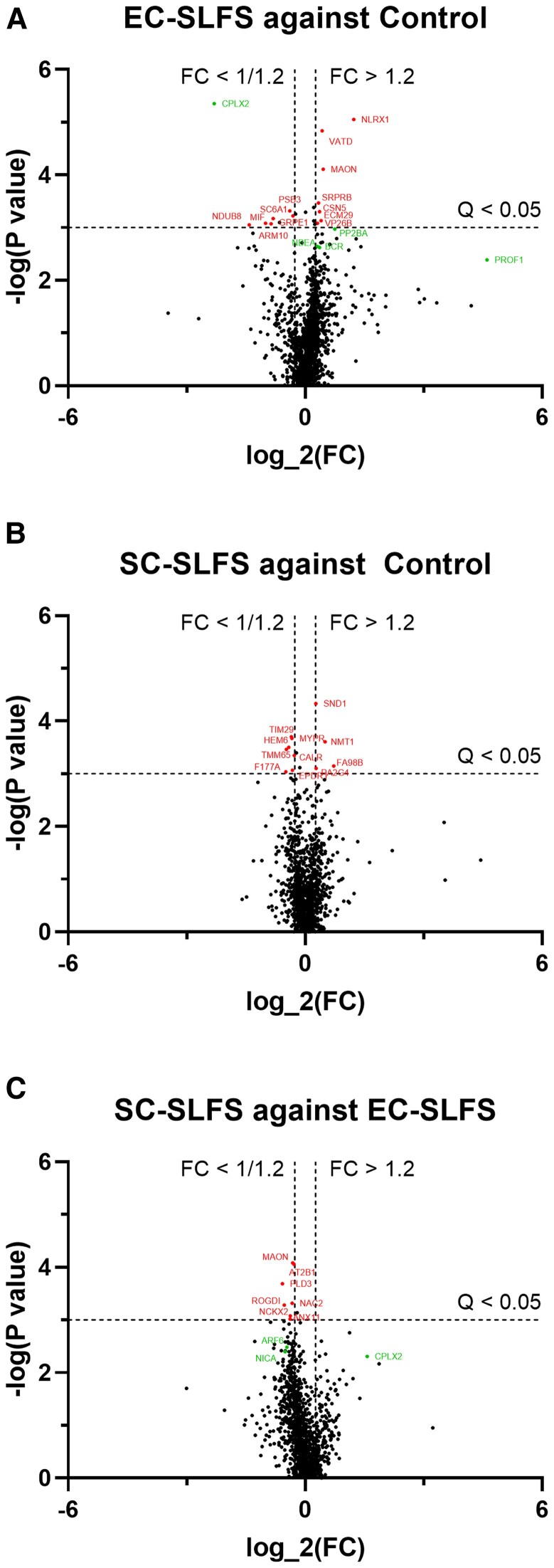
Candidate PRPs that are differentially expressed during LTD maintenance. SC-SLFS, EC-SLFS, and control samples (*n* = 3 per group) were analyzed using SWATH-MS, a form of quantitative shotgun proteomics analysis, to identify potential PRPs. Volcano plots representing comparisons of A) EC-SLFS against control, B) SC-SLFS against control, and C) SC-SLFS against EC-SLFS are presented here. Horizontal dotted lines represent the statistical significance threshold adjusted for false discovery rate (*Q* < 0.05). Vertical dotted lines represent a fold change threshold of 1.2 × in either direction as a point of convenient reference. Each point represents a protein identified during data analysis (black points). Proteins that are statistically significant or near the statistical significance threshold (*Q* < 0.07) and have known postsynaptic localization via GO term annotations are named (red points). A last manual filtering step based on previously reported protein function yields 5 most likely candidate proteins (green points). FC represents fold change. Table 1 provides further details on proteins that pass the initial filter (both red and green points).

We have thus far found that several common LTD-related PRPs are not critically involved in SLFS-induced LTD at EC-CA2 synapses (37, 40). To obtain additional candidate proteins that could be PRPs required for EC-CA2 LTD, we chose a shotgun proteomics approach to identify proteins that are differentially expressed as a result of SLFS at CA2 synapses. Samples were obtained by manually dissecting out the CA2 from slices that were either untreated, EC-SLFS or SC-SLFS in similar fashion as samples prepared for proBDNF ELISA. SWATH-MS (see Materials and methods) was the mass spectrometry method of choice for sample analysis. A total of 1,385 proteins were detected, of which 42 were significantly different (*Q* < 0.05) in at least one of the conditions. PRPs, by definition, must be able to take part in the STC process via interacting with synaptic tags present at postsynaptic sites. Thus, we filtered the hits for synaptic proteins based on their cellular localization as reported in their GO terms. Filtering by statistical significance, fold change (at least 1.2×) and cellular localization resulted in six hits, of which five are unlikely to be PRPs, being various proteins that regulate ion and neurotransmitter movement across the membrane, or being mainly localized to the presynaptic site. The last protein, CPLX2, has previously been reported to have a positive regulatory role in LTP, which is consistent with it being downregulated (almost 80% less in EC-SLFS versus control) to support LTD maintenance (42). To obtain a few more candidate proteins for testing, we selected proteins close to statistical significance (*Q* < 0.07) that are still located within synapses, resulting in a total of 27 hits (Table 1; Fig. 5, red points), of which five are of interest after manual filtering based on protein function and convenience of verification (Table 1, proteins marked with *; Fig. 5, green points). These are our remaining candidates of interest: CPLX2, ARF6, NICA, PP2BA, and PROF1. Volcano plots of the proteomics data are presented in Fig. 5 (comparing EC-SLFS against control, Fig. 5A; SC-SLFS against control, Fig. 5B; SC-SLFS against EC-SLFS, Fig. 5C). We also detected CA2-specific proteins such as RGS14 and STEP, confirming that the samples obtained were accurately dissected.

**Table 1.** Filtered candidate PRPs that are differentially expressed during LTD maintenance.

Protein name	UniProt ID	Fold change	*Q*-value
*SC-SLFS against EC-SLFS*			
Plasma membrane calcium-transporting ATPase 1	G5E829	0.821	0.0271
Protein rogdi homolog	Q3TDK6	0.692	0.0479
Sodium/calcium exchanger 2	Q8K596	0.795	0.0479
Annexin A5	P48036	0.687	0.0567
Synaptotagmin-1	P46096	0.739	0.0637
60S acidic ribosomal protein P0	P14869	1.326	0.0637
Vesicle-associated membrane protein 7	P70280	0.789	0.0637
ADP-ribosylation factor 6 (ARF6)*	P62331	0.725	0.0655
Nicastrin (NICA)*	P57716	0.704	0.0657
Complexin-2 (CPLX2)*	P84086	2.975	0.0677
Synapsin-2	Q64332	0.798	0.0677
Prohibitin-2	O35129	0.808	0.0677
Brevican core protein	Q61361	0.827	0.0677
			
*EC-SLFS against unstimulated controls*			
Complexin-2 (CPLX2)*	P84086	0.202	0.0096
NLR family member X1 (NLRX1)^#^	Q3TL44	2.341	0.0096
Sodium- and chloride-dependent GABA transporter 1	P31648	0.569	0.0479
COP9 signalosome complex subunit 5	O35864	1.289	0.0479
Protein phosphatase 3 catalytic subunit alpha (PP2BA)*	P63328	1.683	0.0523
Protein rogdi homolog	Q3TDK6	1.319	0.0627
Neurobeachin (NBEA)^#^	Q9EPN1	1.286	0.0637
Breakpoint cluster region protein (BCR)^#^	Q6PAJ1	1.238	0.0637
Ezrin	P26040	1.410	0.0655
Profilin-1 (PROF1)*	P62962	24.319	0.0662
Spectrin beta chain	Q68FG2	1.203	0.0665
Alpha-synuclein	O55042	1.284	0.0673
Tropomodulin-2	Q9JKK7	1.241	0.0677
			
*SC-SLFS against unstimulated controls*			
Alpha-internexin	P46660	0.808	0.0528
Neurofilament light polypeptide	P08551	0.813	0.0528
Ras-related protein Rab-21	P35282	0.794	0.0655

This table lists all proteins identified during SWATH-MS analysis that have statistically significant differential expression (*Q* < 0.05), or are close to the statistical threshold (Q < 0.07) and are known to be localized postsynaptically as annotated by GO terms. Manual curation according to known protein functions yields five top candidate PRPs that were further verified via western blot (indicated by *). Other proteins of potential interest with less relevant protein functions are highlighted but not further investigated in this study (indicated by ^#^).

**Fig. 6.**
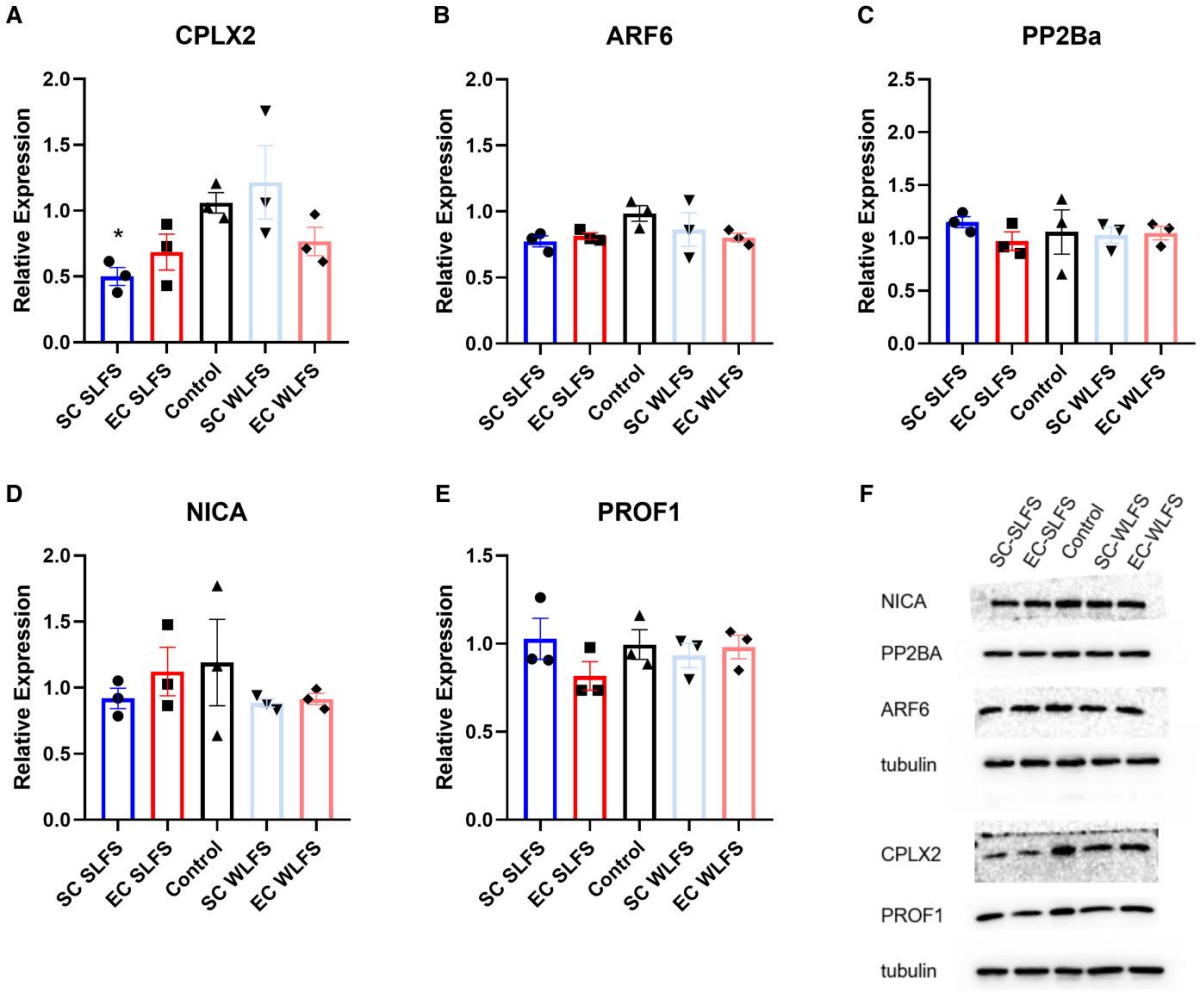
Verification of differentially expressed proteins yields CPLX2 as a strong candidate PRP for CA2 LTD. Western blot was performed to quantify the five candidate proteins, A) CPLX2, B) ARF6, C) PP2Ba, D) NICA, and E) PROF1, to verify the mass spectrometry results. Protein quantification was performed between five sample conditions (*n* = 3 per condition): SC-SLFS (blue bar), EC-SLFS (red bar), unstimulated slices (black bar), slices collected 30 min after WLFS application to SC-CA2 synapses (SC-WLFS, light blue bar), and slices collected 30 min after WLFS application to EC-CA2 synapses (EC-WLFS, light red bar). Relative expression is calculated as a ratio of protein fluorescence signal over tubulin fluorescence signal for each sample. F) Representative blots for all five candidate proteins. Tubulin serves as a loading control. *Asterisks indicate differences of statistical significance (*P* < 0.05). Error bars indicate ± SEM. *n* refers to biological replicates.

To verify the differential expression reported via proteomics-based methods, we sought to quantify in a more targeted manner the expression levels of each of these proteins via Western blot. For each protein, samples are collected in the following conditions: unstimulated controls, EC-SLFS, SC-SLFS, 30 min after WLFS stimulation at EC-CA2 (EC-WLFS), and 30 min after WLFS stimulation at SC-CA2 (SC-WLFS). The WLFS stimulated samples would contain synaptic tag proteins but not PRPs, while the SLFS stimulated samples are expected to have both tags and PRPs. Of the candidate proteins tested, only CPLX2 yielded significantly lower expression of the protein in SC-SLFS samples compared with control (Welch's ANOVA W = 5.763, *P* = 0.0431; Games–Howell's post hoc test *P* = 0.0263; Fig. 6A). There is also a negative trend when comparing EC-SLFS against control, SC-SLFS against SC-WLFS, and EC-WLFS against control, although none of these comparisons are significant. Of the other proteins tested (ARF6, Fig. 6B; PP2Ba, Fig. 6C; NICA, Fig. 6D; PROF1, Fig. 6E), none show significantly different expression levels after stimulation. Hence, we have strong evidence supporting the idea that the reduced expression of CPLX2 plays a significant role in the maintenance of LTD in the CA2.

## Discussion

The findings within this paper demonstrate that CA2 hippocampal pyramidal neurons are plastic against LTD-inducing electrical stimulus, in contrast to their LTP-resistant nature. LTD can be induced at both EC-CA2 and “less plastic” SC-CA2 synapses. LTD at both synapses shares properties, both being NMDA receptor, protein synthesis, and p75^NTR^-dependent in a similar fashion as the classical SC-CA1 synaptic population (29–31). However, the two synaptic populations differ in the ease of LTD induction. “Ease” of induction here could refer to two possible measures: consistency of induction or the stimulation magnitude threshold required for induction. Intriguingly, both synapses could be considered being difficult to induce LTD in, depending on which measure was taken. SLFS induction of LTD in EC-CA2 synapses was inconsistent, with only ∼67% of experiments ending in successful induction of LTD, as compared to more than 85% (only one case that returned close to baseline, Fig. 1D) in SC-CA2. This inconsistency corroborates with earlier studies that compared LTD (in a patch clamp setup) in CA2 with CA1, where it is more consistent (10). Note that Zhao et al. (10) used a 2-Hz stimulation on rat tissue under patch clamp; hence, comparisons with this earlier study must be cautious. On the other hand, there is a lower threshold for LTD expression in EC-CA2 synapses, which express early LTD after WLFS, as compared to SC-CA2 synapses, which do not. These distinctions in plasticity properties not only confirm what is well known—that EC-CA2 and SC-CA2 synapses are greatly distinct, but also that EC-CA2 synapses themselves have different plasticity than other nearby synaptic populations (17).

Distinctions in plasticity properties often manifest as a result of structural or biochemical changes between the synaptic populations. A decent amount of literature discussing biochemical distinctions in CA2 pyramidal neuron synapses exist, and these provide some plausible explanations for the plasticity differences we observe in SC-CA2 synapses. Note that these biochemical distinctions are typically discussed in the context of explaining LTP-resistance observed at SC-CA2 synapses. It is known that CA2 synapses have enhanced calcium-buffering and extrusion capacities (16). These capacities are caused by increased expression of calcium-interacting proteins such as RGS14 which are necessary for blocking LTP (14, 15). Since NMDAR-dependent LTD involves small but sustained calcium transients in the postsynaptic space during its initiation, these calcium-buffering capabilities could have suppressive effects on these transients that would be especially prominent during weaker stimulations (i.e. WLFS). CA2 neurons also have increased expression of STEP, an ERK inhibitor, while induction of LTD is ERK dependent (37, 43). These are some mechanisms that could account for the higher threshold for LTD inductance within SC-CA2 synapses. The inconsistency in the maintenance of LTD at EC-CA2 synapses, on the other hand, remains a mystery that requires further investigation into the properties of these synapses. Considering the differences in plasticity properties between the two sets of synapses, it is likely that there are distinct explanatory biochemical mechanisms present at EC-CA2 synapses.

Our study also reveals another intriguing difference between the two synaptic populations—that they could involve different signaling pathways to achieve LTD even with the same plasticity-inducing stimulus. We demonstrate that only maintenance of LTD at SC-CA2 synapses is dependent on proBDNF. Strangely, late LTD in EC-CA2 synapses is still dependent on p75^NTR^ but not its interaction with proBDNF. It is possible that the necessity for p75^NTR^/proBDNF interaction is only during the induction phase of LTD. Another study has shown that the introduction of BDNF propeptide during LTD induction enhances the degree of synaptic depression (36), which supports this possibility since this propeptide is able to interact with p75^NTR^. Timing-wise, p75^NTR^ could have an influence during LTD induction, although our data only prove its necessity during the maintenance phase. It is also possible that some other small molecule is responsible for interacting with p75^NTR^, which is a reasonable possibility since p75^NTR^ is a pan-neurotrophin receptor (44, 45). It should be noted here that p75^NTR^ has been detected within the hippocampus in both protein and mRNA form, although numerous recent transcriptomics studies suggest that this expression is low and difficult to detect (30, 46–49). proBDNF has also been shown to be secreted by hippocampal neurons, although this idea has been somewhat challenged more recently (50, 51). Our data support the established stance on both proteins by demonstrating expression in the CA2.

Furthermore, the proteomics data suggest that there is differential expression of CPLX2 after SLFS. CPLX2 was initially found to be SNARE complex binding proteins that are heavily expressed within the presynaptic space. CPLX2 is typically known for its function in regulating neurotransmitter release via modulating vesicle fusion at the presynaptic membrane (52). Postsynaptically, CPLX2 has been found to be important for LTP via its role in moderating α-amino-3-hydroxy-5-methyl-4-isoxazolepropionic acid (AMPA) receptor exocytosis (42). While our protein quantification methods did not agree on the synaptic population in which CPLX2 has statistically significant differential expression, they both provide evidence for a decrease in CPLX2 expression during the maintenance phase of LTD. This decrease in expression is consistent with the known functions of CPLX2, which promotes LTP. The involvement of CPLX2 in AMPA receptor exocytosis further strengthens the possibility of it being a PRP, as regulating AMPA receptor availability at the synapse in the long term is a core function in other proven PRPs such as PKMζ (53).

Interestingly, proBDNF and CPLX2 both have significant presynaptic presence, which could be another cause for the different plasticity properties we observe between EC-CA2 and SC-CA2 synapses. It is known that presynaptic BDNF from the CA3 is required for some forms of LTP in the CA1 (54). BDNF expression appears to be lower in the entorhinal cortex as compared to the CA3, which corresponds well with our ELISA data (55). CPLX2 is also known to have a presynaptic role in LTP, including promoting vesicle fusion during calcium events (56–58). It is entirely possible that the differences we are observing here indicate presynaptic heterogeneity, since our protein analysis methods include both presynaptic and postsynaptic components. It is also possible for the presynaptic–postsynaptic distribution of these proteins to differ between the two synaptic populations. These are important considerations for future studies looking to understand these heterogeneous synaptic populations. Alternate splicing of BDNF is another mechanism that could result in its heterogeneous distribution within a neuron (59). Given screening-based evidence that abundance of difference splice variants varies between different hippocampal subregions, it is possible that the specific mixture of splice variants in the CA2 could account for this unequal distribution (48). Here, we must also raise the possibility that these observed difference in proBDNF expression could instead be a consequence of the different synaptic properties of the two synaptic populations. EC stimulation is known to produce stronger excitation in CA2 dendrites as compared to SC stimulation (17, 60). The stronger excitation could drive cleavage of proBDNF to its mature form, although the levels of neuronal activity discussed in these earlier studies are much stronger (61, 62).

The idea that a protein could serve as a PRP via a reduction in its expression has many considerations for further study. Note that PRPs are typically depicted as having an increase in expression postinduction and the STC hypothesis describes the capture process as an interaction between newly synthesized PRPs and synaptic tags (9). How could a synaptic tag capture a PRP when its presence is reduced? One possibility could involve said PRP being constitutively present at synapses and performing an inhibitory function that prevents the stabilization of the synaptic tag. Consequently, its reduction allows for what is observed as STC. In this case, CPLX2 is known to cause AMPAR exocytosis in the postsynaptic space (42). Hence, its reduced expression could reduce AMPAR exocytosis that results in reduced synaptic efficacy. Other biochemical changes could then serve as part of the synaptic machinery to capture the change in expression of CPLX2, perhaps with some similarity to the PKMζ system. An important caveat must be made here: it is entirely possible that CPLX2 is simply being regulated by some other proteins that are increased in expression during LTD. This could include some of the mass spectrometry hits as the filtering strategy used in this study does occlude potential candidate proteins with undiscovered postsynaptic localizations. For instance, NLRX1 is a mitochondrial protein that has significant differential expression (Q < 0.05) and a large fold change (Table 1). Since mitochondrial proteins have recently been associated with plasticity functions in the CA2, NLRX1 could very well have potential novel PRP functionality or interaction (48). Other potential proteins are highlighted in Table 1 (proteins marked with ^#^). As more information about synaptic and plasticity proteins is unveiled, these proteomics results could be revisited to provide new candidates for investigation in future studies.

## Conclusion

Our study demonstrates and characterizes LTD in hippocampus CA2 pyramidal neurons. CA2 LTD shares basic properties with LTD at SC-CA1 synapses, but is heterogeneous between SC-CA2 and EC-CA2 synapses. These populations differ in LTD induction threshold and consistency and differ biochemically in terms of their dependency on proBDNF and CPLX2, which possibly play the role of PRPs at these synapses. Further investigation into the role of CPLX2 as a PRP which exerts influence over the synaptic tag via downregulation could aid in the refinement of the STC hypothesis. Elucidating the differences and mechanisms responsible for the creation and maintenance of such distinctions between EC-CA2 and SC-CA2 synapses would likely enhance our understanding of hippocampal circuitry and the complex role of CA2 within these circuits.

## Materials and methods

All experimental animal protocols (protocol numbers: R20-0168, R21-0802, and R24-0227) adhere strictly to regulations from and were approved by the Institutional Animal Care and Use Committees (IACUC) at the National University of Singapore. Detailed methods’ descriptions are given in Supplementary File 3.

## Supplementary Material

pgaf241_Supplementary_Data

## Data Availability

All data generated or analyzed during this study are included in the manuscript and supplementary information.
