# Supplementary material for: Differential expression of long-term depression, and synaptic tagging and capture in mouse hippocampal area CA2 synapses
Source: PNAS Nexus. 2025 Jul 29;4(8):pgaf241. doi: 10.1093/pnasnexus/pgaf241 (PMC12344489; doi:10.1093/pnasnexus/pgaf241)

Raw Western Images

Differential Expression of Long-Term Depression, Synaptic Tagging and Capture in Mouse Hippocampal Area CA2 Synapses

Sample Set 1

NICA

PP2BA

tubulin

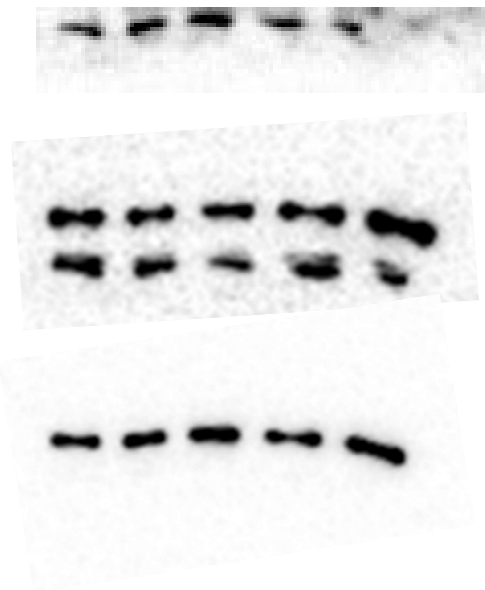

tubulin

ARF6

PROF1

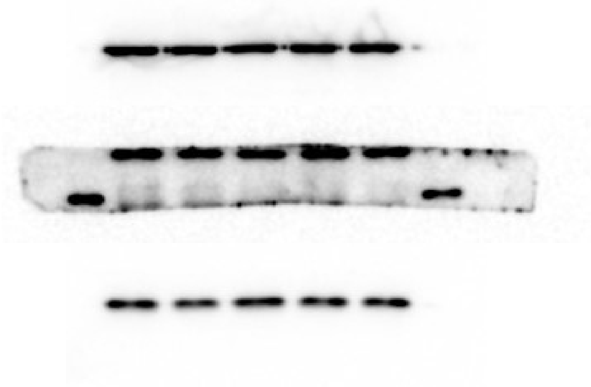

CPLX2

tubulin

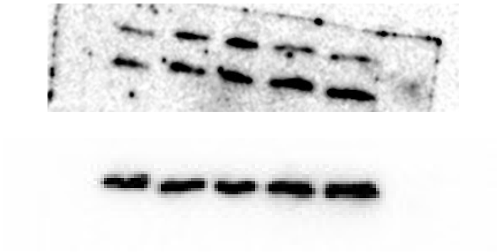

Sample Set 2 (Representative, Fig 6F)

NICA

PP2BA

ARF6

tubulin

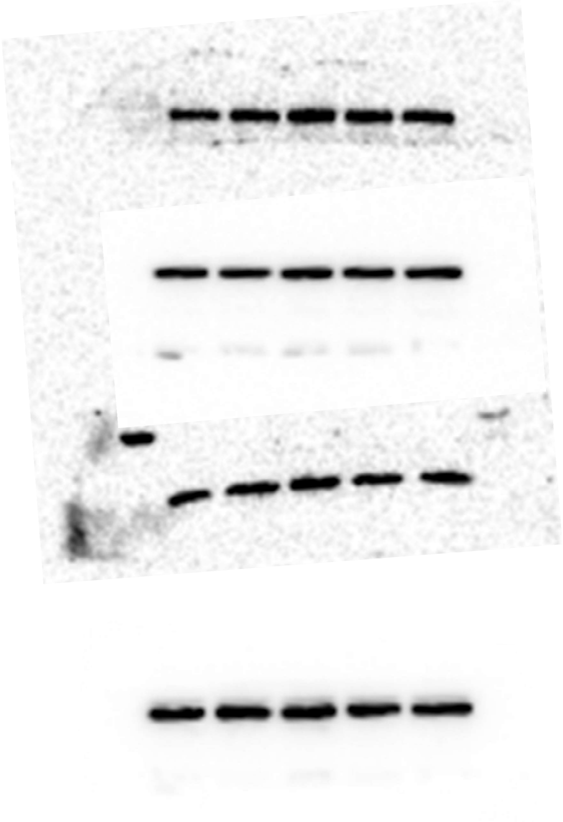

tubulin

CPLX2

PROF1

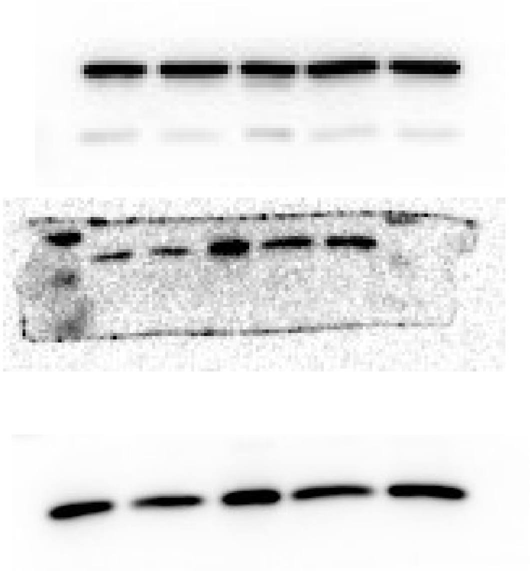

Sample Set 3

CPLX2

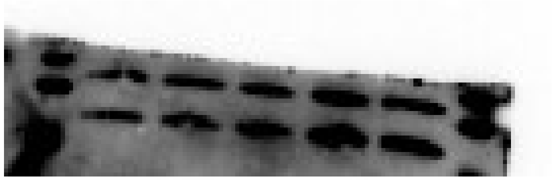

tubulin

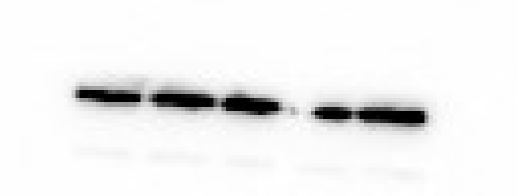

PROF1

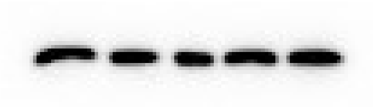

tubulin

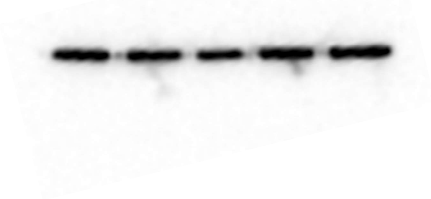

NICA

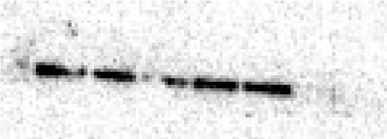

PP2BA

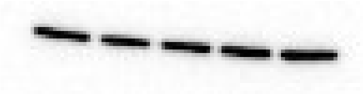

ARF6

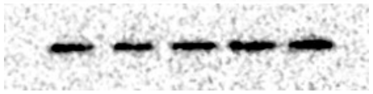

tubulin

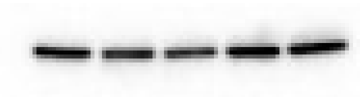

Supplement: pgaf241_Supplementary_Data [file pgaf241_supplementary_data.zip › PNASNEXUS-PNASNEXUS-2025-00288R-s02.pdf]
