# Supplementary material for: Differential expression of long-term depression, and synaptic tagging and capture in mouse hippocampal area CA2 synapses
Source: PNAS Nexus. 2025 Jul 29;4(8):pgaf241. doi: 10.1093/pnasnexus/pgaf241 (PMC12344489; doi:10.1093/pnasnexus/pgaf241)
Supplement: pgaf241_Supplementary_Data [file pgaf241_supplementary_data.zip › PNASNEXUS-PNASNEXUS-2025-00288R-s03.docx]

**Supplementary File 3 – Materials and Methods**

*Animals*

A total of 82 5-7 weeks old male C57BL/6 mice and 17 5-7 weeks old p75^NTR^ knockout (p75KO) mice were used throughout this study. p75KO mice were generated by backcrossing p75^NTR^ KO mice (exon III) from Jackson Laboratory (Bar Harbor, Maine, US) to the C57BL/6 background for at least 10 generations (1). Animals were group housed in standard cages under 12-h light/dark cycles with *ad libitum* access to food and water. All experimental animal protocols (protocol numbers: R20-0168, R21-0802, R24-0227) adhere strictly to regulations from and have been approved by the Institutional Animal Care and Use Committees (IACUC) at the National University of Singapore.

*Electrophysiology*

The protocol for long-duration *in vitro* slice electrophysiology experiments have been reported elsewhere in detail (2). Briefly, mice involved in electrophysiology experiments were anesthetized using CO_2_ and sacrificed by decapitation. Their brains were swiftly removed and transferred to oxygenated 4 °C artificial cerebrospinal fluid (aCSF). aCSF consisted of the following compounds (in mM): 124 NaCl, 3.7 KCl, 1 MgSO_4_.7H_2_O, 2.5 CaCl_2_.2H_2_O, 1.2 KH_2_PO_4_, 24.6 NaHCO_3_, and 10 D-glucose. aCSF was kept oxygenated throughout the experiments through perfusion of a 95% oxygen-5% CO_2_ gaseous mixture (carbogen) at a flow rate of 16 L/h. 400 μm thick transverse hippocampal slices were collected from the excised brains using a manual tissue chopper. Slices were then incubated at 32 °C in an interface chamber (Scientific Systems Design, Ontario, Canada) for two hours prior to experiments. aCSF flow rate was set to 1 mL/min. All reported electrophysiology experiments below are two pathway experiments, consisting of two stimulating electrodes and a recording electrode. Stimulating electrodes for the EC-CA2 pathway were placed within the *stratum lacunosum-moleculare* towards the CA1, while those for the SC-CA2 pathway were placed within the *stratum radiatum* towards the CA3. The recording electrode was placed within the CA2 distal dendritic layer (Figure 1A). Signals from these recordings were amplified via a differential amplifier (Model 1700; AM Systems, Sequim, Washington, US) and subsequently digitized using a CED 1401 analog-to-digital converter (Cambridge Electronic Design, Cambridge, UK). Monopolar, lacquer-coated stainless steel electrodes (5 MΩ; AM Systems) were used in all experiments. After incubation, an input-output curve (afferent stimulation against fEPSP slope) was plotted to determine the maximal output. The strength of experimental stimulation was set to 40% of this maximum. Test stimuli, consisting of four 0.2 Hz biphasic constant-current pulses (0.1 ms/polarity), were applied every 5 minutes throughout the experiment, except during other electrical manipulations. The fEPSP slopes were monitored online using custom software (Intracell). Late-LTD was induced via strong low frequency stimulation (SLFS), which consists of 900 bursts (consisting of 3 pulses at 20 Hz) at 1 Hz. Early-LTD was induced via weak low frequency stimulation (WLFS), which consists of 900 pulses at 1 Hz. Test stimuli were delivered for four hours after the first LTD inducing stimulus. Experiments with unstable or erratic control pathway recordings were excluded from the study. Note that N numbers reported here refer to slice replicates.

*Pharmacology*

For field electrophysiology experiments that require pharmacological manipulations, drugs were added to the aCSF supply and perfused throughout the setup. For acute manipulations, drugs were made available for 30 min before and after the LTD stimulation to be affected, for a total duration of 1 hour. For experiments on LTD maintenance, drugs were added from 30 min after LTD induction until the end of the experiment. Protein synthesis inhibitor emetine was prepared by first dissolving emetine dihydrochloride (Sigma Aldrich, Darmstadt, Germany) in dH_2_O to form a 20 mM stock solution, then diluting in aCSF prior to experiments for a 20 μM experimental solution. NMDA receptor antagonist AP5 (Tocris, Minneapolis, Minnesota, US) was first dissolved in DMSO to form a 50 mM stock solution, then diluted in aCSF for a 50 μM experimental solution. TrkB-Fc chimeric protein (Cat # 688-TK, R&D Systems, Minneapolis, Minnesota, US) was prepared fresh every experiment by dissolving the lyophilized form in dH_2_O to form a 1 μg/mL solution. ERK inhibitor U0126 (Promega, Madison, Wisconsin, US) was stored after dissolving in water as a 1 mM stock solution, then diluted in aCSF immediately prior to experiments for a final concentration of 1 μM. MAPK14 inhibitor SB203580 (Tocris) was dissolved in DMSO to form a 20 mM stock solution, then diluted in aCSF to 20 μM for experimental use. All stock solutions were stored at -20 °C.

*Mass Spectrometry*

Hippocampus slices were prepared in the same manner as for electrophysiology experiments. Control (i.e. unstimulated) slices were collected immediately after incubation, while SLFS stimulated slices were collected 1 hour after SLFS was delivered to either EC-CA2 or SC-CA2 synaptic pathways respectively. The CA2 region was dissected out of all slices, which were immediately snap frozen and stored at -80 °C. Slices were then sent to the Protein and Proteomics Center (PPC; Department of Biological Sciences, National University of Singapore) for tissue processing and proteomics analysis on a commercial basis. All subsequent steps for mass spectrometry analyses were performed by PPC.

Protein extraction and digestion from tissue was performed using S-TRAP Micro (Protifi, Fairport, New York, US) according to manufacturer instructions. Total peptide quantification was performed using Pierce Quantitative Colorimetric Peptide Assay (Thermo Fisher Scientific, Waltham, Massachusetts, US) to normalize peptide concentrations across samples for subsequent liquid chromatography mass spectrometry (LCMS) analysis. Synthetic iRT peptides (Biognosys, Schlieren, Switzerland) were spiked in each sample at 10% final concentration for retention time alignment. Sequential window acquisition of all theoretical fragment ion spectra (SWATH)-MS analysis was performed using the Waters ACQUITY UPLC M-Class System (Waters, Milford, Massachusetts, US) coupled with TripleTOF 6600 system (SCIEX, Framingham, Massachusetts, US). Sample peptides were first loaded onto a nanoEase M/Z Symmetry C18 trap column (5 μm 100 Å 180 μm x 20 mm, Waters) before separation through a nanoEase M/Z Peptide BEH C18 analytical column (1.7 μm 130 Å 75 μm x 250 mm, Waters). Mobile phase A (0.1% formic acid) and mobile phase B (0.1% formic acid in acetonitrile) were used to create the elution gradient. This elution gradient was formed as follows: mobile phase B 5-15% for 60 min, 15-30% for 60 min, 30-90% for 3 min, maintained at 90% for 20 min, 90-5% for 2 min, then sustained at 5% for 15 min (300 nL/min flow rate). Precursor ions were acquired across a range of 400 – 1600 m/z with 50 ms accumulation time for each spectrum. Fragmentation information was collected using a setup of 100 variable SWATH windows across 400-1200 m/z precursor mass range. Each SWATH window was acquired in high sensitivity mode for 30 ms across 100-1800 m/z, with rolling collision energy enabled with 5 eV spread.

SWATH data were analyzed using the DirectDIA workflow in Spectronaut 18 software (Biognosys). Pulsar library search was performed using Trypsin/P against a database consisting of the UniProt *Mus musculus* reference proteome (UP000000589, 2023_01 release), spiked with common contaminant proteins (cRAP). Methyl methanethiosulfonate (MMTS) was specified as a fixed modification, while acetyl (protein N-term) and oxidation (M) were permitted variable modifications. The false discovery rate (FDR) was set at 0.01. Data independent acquisition-based data extraction was performed with dynamic retention time window and ion mass tolerance extraction. Quantification was performed from the summation of the top 10 proteotypic peptides. The Q-value cut-off was set to 0.05. Differential abundance was determined using unpaired t-tests. A global normalization strategy on median was employed.

*Western Blot*

Samples were collected in a similar fashion as those sent for mass spectrometry analysis, with the addition of WLFS stimulated samples. These slices differ in that they were dissected and collected only 30 min after WLFS to either EC-CA2 or SC-CA2 synapses. Proteins were extracted from samples via physical homogenization in Tissue Protein Extraction Reagent (T-PER, Thermo Fisher Scientific) supplemented by protease and phosphatase inhibitors (HALT, Thermo Fisher Scientific). Samples were then centrifuged at 4 °C for 5 min at 10000 rpm. Protein concentrations were measured using Bradford assay, then samples diluted to equal concentration before heating at 95 °C for 10 min with sample buffer. Processed samples were then loaded onto SDS-polyacrylamide gels for separation, followed by transfer onto PVDF membranes via wet transfer. Membranes were blocked using 5% w/v dry milk in 1x TBST then immunoblotted with one of the following antibodies: rabbit anti-profilin 1 (PROF1, 1:1000; Cat # 3237S, Cell Signaling Technology, Danvers, Massachusetts, US), rabbit anti-complexin 2 (CPLX2, 1:1000; Cat # MBS9201735, MyBioSource, San Diego, California, US), mouse anti-ADP ribosylation protein 6 (ARF6, 1:1000; Cat # sc-7971, Santa Cruz Antibodies, Dallas, Texas, US), mouse anti-serine/threonine phosphatase 2B catalytic subunit alpha (PP2BA, 1:1000; Cat # sc-17808, Santa Cruz Antibodies), rabbit anti-nicastrin (NICA, 1:1000; Cat # 5665S, Cell Signaling Technology) or mouse anti-tubulin (1:20000; Cat # T9026, Sigma Aldrich). They were then incubated with their respective peroxidase-conjugated secondary antibodies which were either anti-mouse (1:3000; Cat # A4416, Sigma Aldrich) or anti-rabbit (1:3000; Cat # 170-6515, Bio-Rad, Hercules, California, US). Fluorescent signals were then generated using SuperSignal West Pico Chemiluminescent Substrate (Thermo Fisher Scientific) for imaging. Quantification was performed via densitometric measurement of blots using ImageJ (NIH). The densitometric measurements were normalized to that of tubulin, which served as a loading control. Reported replicates were from different biological samples.

*ELISA*

proBDNF levels in mouse hippocampus samples were observed using a commercially available proBDNF human/mouse ELISA kit (Cat # BEK-2217, Biosensis, Thebarton, Australia), operated in accordance with the manufacturer’s protocol. Samples were collected and prepared in a similar fashion as those used in Western blot. Briefly, provided standards and samples of equal protein concentration were added to wells coated with proBDNF primary antibody. Samples were then washed out and replaced with a biotinylated anti-proBDNF detection antibody. Streptavidin-HRP conjugate was then added to the wells. Lastly TMB solution, followed by a TMB stop solution, was added to yield a yellow coloration to each sample, which had intensity relative to the proBDNF concentration. The colorimetric reading was then obtained using a spectrophotometer which reads absorbance at 450 nm. The reading was then compared against a standard curve to obtain the sample concentration. Reported replicates were from different biological samples.

*Statistical Analysis*

Statistical analysis for all non-proteomics experiments were performed using GraphPad Prism 8.0 (GraphPad, Boston, Massachusetts, US). All data was represented as mean +/- standard error of mean (SEM). For field electrophysiology experiments, fEPSP values were represented as a percentage of the average fEPSP slope value during baseline recording. Wilcoxon signed rank test (Wilcox test) was used to compare within one group (e.g. baseline against experimental values of the same synaptic population), while Mann-Whitney *U* test (*U* test) was used when data was compared between groups (e.g. experimental pathway against control pathway). For biochemistry experiments, Welch one-way ANOVA with Games-Howell’s post hoc test was used to compare between conditions as homogeneity of variance is uncertain. *P* < 0.05 was considered the threshold for differences to be considered statistically significant.

**References**

1. K. F. Lee *et al.*, Targeted mutation of the gene encoding the low affinity NGF receptor p75 leads to deficits in the peripheral sensory nervous system. *Cell* **69**, 737-749 (1992).

2. M. S. Shetty *et al.*, Investigation of Synaptic Tagging/Capture and Cross-capture using Acute Hippocampal Slices from Rodents. *J Vis Exp* 10.3791/53008 (2015).
