# Supplementary figures and images for: Differential expression of long-term depression, and synaptic tagging and capture in mouse hippocampal area CA2 synapses
Source: PNAS Nexus. 2025 Jul 29;4(8):pgaf241. doi: 10.1093/pnasnexus/pgaf241 (PMC12344489; doi:10.1093/pnasnexus/pgaf241)

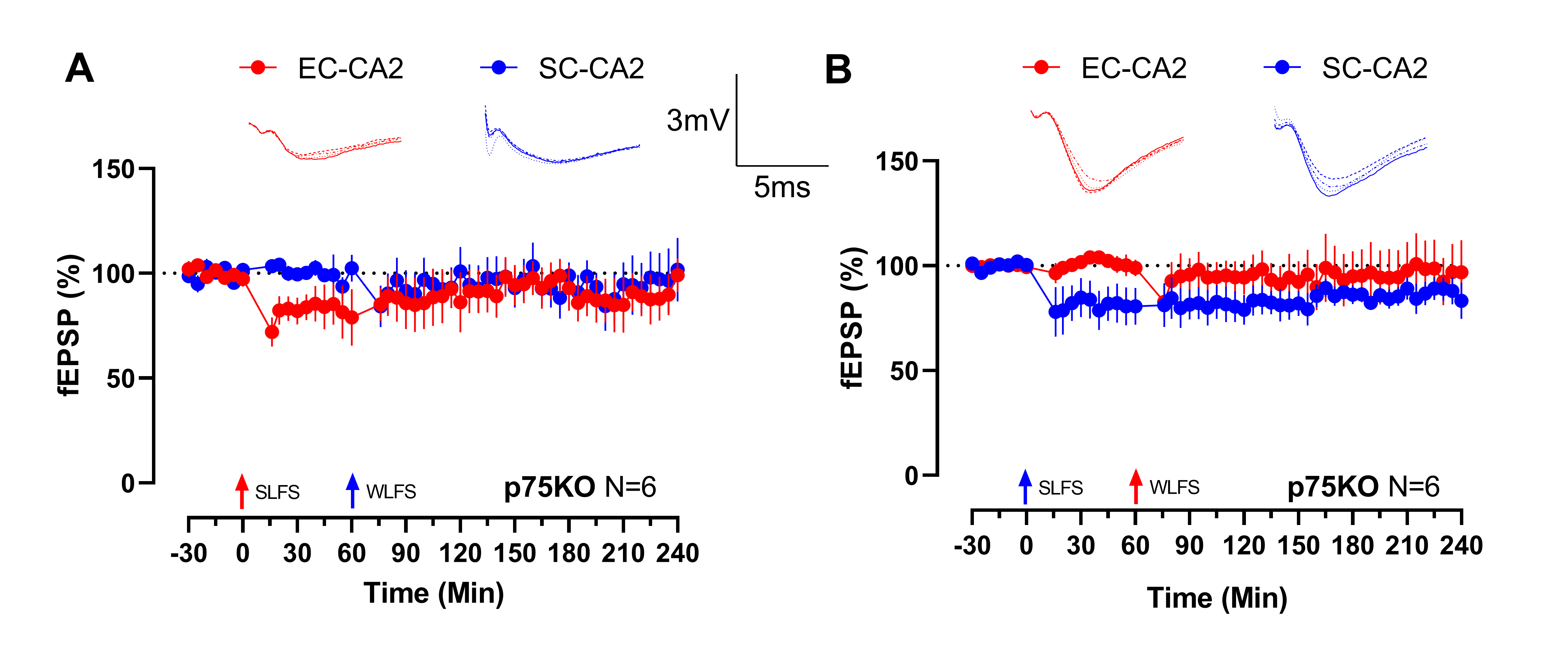

Supplement: pgaf241_Supplementary_Data [file pgaf241_supplementary_data.zip › 'PNASNEXUS-PNASNEXUS-2025-00288R-suppfig01.tif]
